# Simultaneous EUS-FNA Diagnosis and TNM Staging of a Pancreatic Neuroendocrine Tumor in a Patient with an Unrecognized MEN Type 1

**DOI:** 10.1155/2012/619428

**Published:** 2012-10-10

**Authors:** Francesco Ferrara, Carmelo Luigiano, Antonella Maimone, Marco Bassi, Anna Maria Polifemo, Paola Baccarini, Vincenzo Cennamo, Nadia Cremonini, Carlo Fabbri

**Affiliations:** ^1^Unit of Gastroenterology and Digestive Endoscopy, Bellaria and Maggiore Hospitals, AUSL Bologna, Largo Nigrisoli 2, 40133 Bologna, Italy; ^2^Unit of Pathology, Bellaria Hospital, AUSL Bologna, 40139 Bologna, Italy; ^3^Unit of Metropolitan Area Digestive Endoscopy, Bellaria Hospital, AUSL Bologna, 40139 Bologna, Italy; ^4^Unit of Endocrinology, Maggiore Hospital, AUSL Bologna, 40139 Bologna, Italy

## Abstract

We report the case of a woman who, during oncological followup for bronchial carcinoid (diagnosed in 2005), papillary thyroid carcinoma, and bilateral parathyroid adenoma (simultaneously diagnosed in 2007), performed a pancreatic endoscopic ultrasonography with fine needle agobiopsy (EUS-FNA) for a positron emission tomography (PET) suspicion of pancreatic and hepatic lesions; during the procedure, the pancreatic and liver lesions were confirmed, and a peripancreatic lymph node involvement was found, allowing a complete pTNM staging during the same procedure.

## 1. Case

A 48-year-old woman, with no family history of neoplastic diseases, underwent in 2005 to right pulmonary resection for a bronchial carcinoids and was in oncological follow-up for this reason.

In 2007, during a normal physical examination, a 2 cm sized nodule in her left thyroid lobe was palpated. The thyroid function tests were normal. The ultrasonography report revealed a solid hypoechoic nodule measuring 2.5 × 1 cm in the left lobe. A fine needle aspiration was attempted under ultrasound guidance and the final cytodiagnosis was papillary thyroid carcinoma.

Following cytodiagnosis, a total thyroidectomy was performed, but at the time of surgery, during the neck exploration, the patient was found to have bilateral enlargement of parathyroid glands with nodular aspect and all glands were resected. Histology confirmed the diagnosis of thyroid papillary cancer and revealed bilateral parathyroid adenoma.

In September 2008, a biochemical screening revealed high plasma levels of CgA of 2230 (normal 20–150) and serum gastrin (648 pg/mL). She was then referred to our hospital.

A 68Ga-DOTANOC-PET was performed and revealed 3 small areas of hyperaccumulation in pancreatic region and one in the liver.

**Figure 1:**
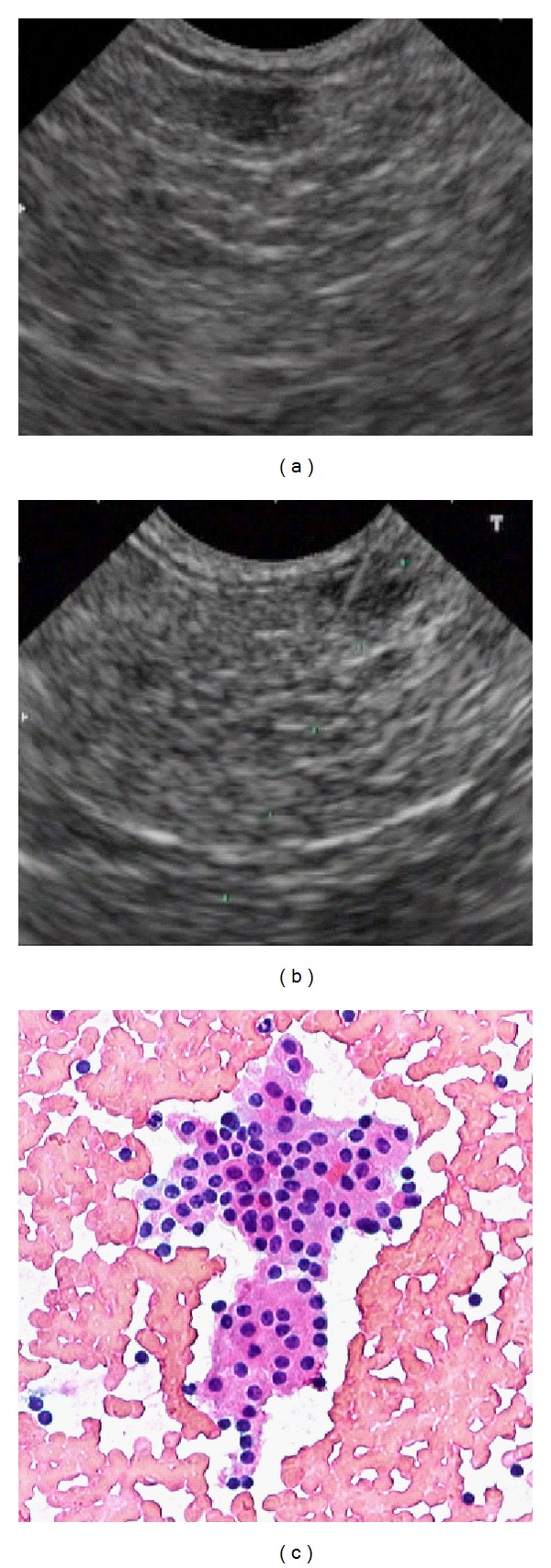
EUS (a), FNA (b), and cytological (c) images of the neuroendocrine pancreatic tumor.

**Figure 2:**
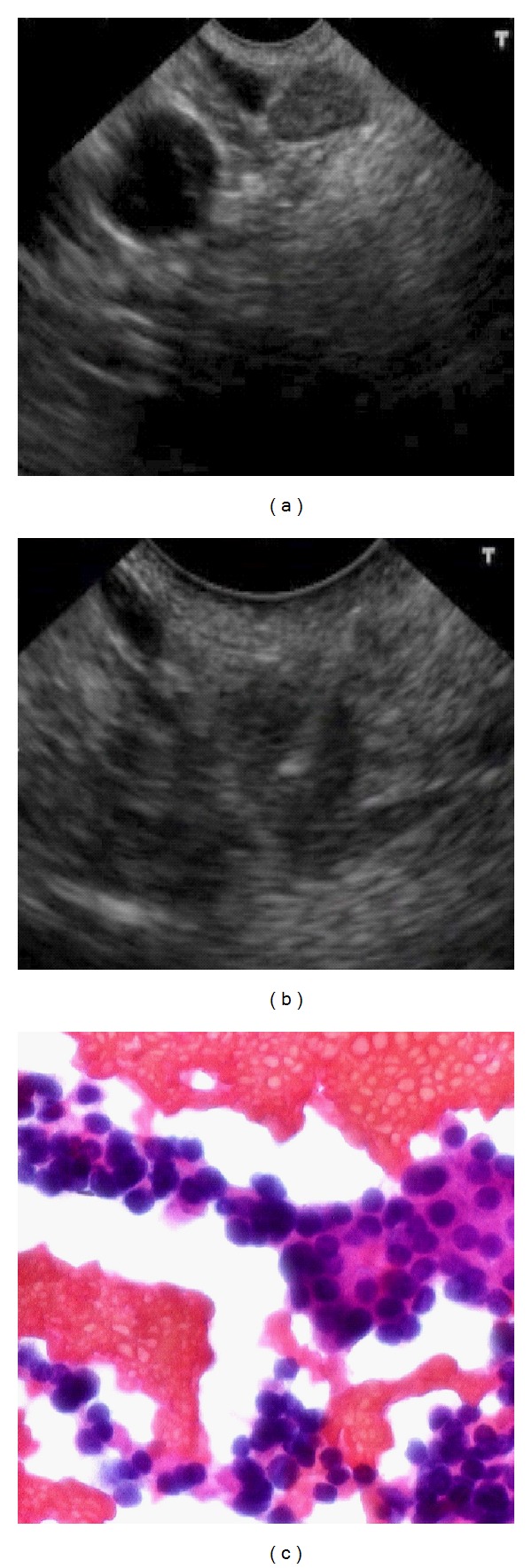
EUS (a), FNA (b), and cytological (c) images of the lymphonodal metastases.

**Figure 3:**
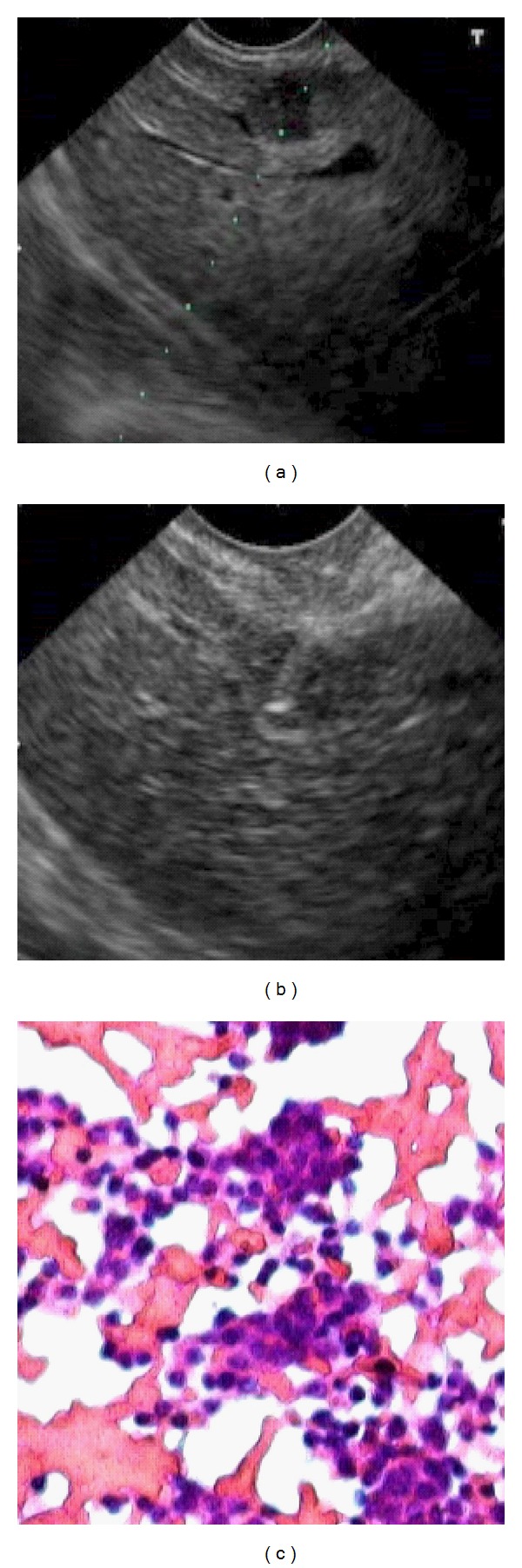
EUS (a), FNA (b), and cytological (c) images of the liver metastases.

For this reason, an EUS was performed and identified 2 nodular hypoechoic lesions of 5 mm with hypervascular Doppler pattern located in the body and in the tail of the pancreas. During the exploration, also a peripancreatic nodal involvement was found and the suspected metastasis (15 mm) of left liver was confirmed. We performed EUS-guided FNA with a 22G and a 25G needle, with on-site cytopathologist, and a Pan-NET (T) tumor (Figure 1) with (N) lymph node (Figure 2) and (M) liver (Figure 3) involvement was diagnosed.

A mutation of the MEN-1 gene was identified and, due to the high level of serum gastrin, a diagnosis of gastrinoma was reached.

She started therapy with octreotide LAR followed by receptor radiometabolic therapy with radiolabelled somatostatin analogues (177 Lu-DOTATATE) and she is presently alive.

## 2. Discussion

Multiple endocrine neoplasia type 1 (MEN-1) syndrome is a rare disease, inherited as an autosomal dominant trait with an estimated prevalence of 0.01–2.5/100000 [1].

MEN-1 syndrome is characterized by parathyroid hyperplasia, neuroendocrine pancreatoduodenal tumors, and pituitary adenomas. Less commonly, MEN-1 patients can develop bronchial, gastrointestinal, and thymic carcinoids, benign thyroid and adrenocortical tumors, lipomas, angiofibromas, skin collagenomas, and ependymomas of the central nervous system [2].

Thyroid disease can be observed in over 25% of MEN1 patients, [3, 4], and it can be detected incidentally during parathyroid surgery.

Only three cases of papillary thyroid cancer combined with MEN1 were reported in the literature and seems that these cases did not correlate to MEN1 [4–6].

Probably for this reason, the diagnosis of MEN1 was not made before.

About 20% of MEN-1 patients succumb to malignant tumors and malignant Pan-NET are unequivocally the most frequent cause of death [7].

Imaging modalities such as CT and MRI have enabled detection of Pan-NETs. Overall sensitivity for CT ranges from 64 to 82%, with lesser sensitivity for tumors <1 cm in size. Similarly, MRI offers superb imaging of the pancreas, with sensitivity of up to 90% in Pan-NETs [8, 9].

Nuclear medicine studies have been developed to target specific receptors on Pan-NET tumor cells with radiolabeled receptor-binding peptides. The most common of these tests is somatostatin receptor scintigraphy (SRS). The sensitivity of SRS for detecting gastrinomas is as high as 75–100%; in contrast, SRS is able to detect insulinomas in approximately only half of the times [10].

Endoscopic ultrasound (EUS) is an extremely valuable tool in the diagnosis and management of these tumors. The important role of EUS in the detection of Pan-NETs was first described in 1992 [11]: EUS demonstrated a sensitivity of 82% and a specificity of 92% in the detection of islet cell tumors in patients whith previously undetected tumors by US and CT. Since then, EUS has been increasingly used in the localization of Pan-NETs [12]. EUS is particularly useful in the detection of smaller insulinomas. The average size of insulinomas at initial diagnosis is 6–10 mm, with 90% of cases under 2 cm [13].

A recent study on 52 patients undergoing EUS for detection of a suspected insulinoma (based on clinical and laboratory findings) reported a sensitivity of 89.5% and accuracy of 83.7% based on surgical findings. The sensitivity of EUS for detection of lesions in pancreatic head, body, and tail was 92.6, 78.9, and 40.0%, respectively [14].

The detection rates for pancreatic gastrinomas by EUS are similar to that of insulinomas, approximately 75–94% [15].

Pan-NETs may be pathologically evaluated by FNA during the EUS examination. Three recent studies reported sensitivities of 61–84% and overall accuracy of up to 92.5% of EUS-FNA in establishing the diagnosis of Pan-NETs [16–18]. Additionally, FNA may detect and confirm the presence of malignant lymph nodes and liver metastases previously unseen on CT imaging [18, 19]. Recently, Lewis et al. [20] further confirmed in 52 patients the high accuracy of EUS-FNA in preoperative assessment in MEN-1, and Barbe et al. [21] established a complementary role for MRI and EUS.

To the best of our knowledge, this is the first report where EUS-FNA has allowed not only the localization but also a complete diagnosis with pTNM staging of Pan-NETs.
